# Serum Neurofilament Light Chain Levels and Myelin Oligodendrocyte Glycoprotein Antibodies in Pediatric Acquired Demyelinating Syndromes

**DOI:** 10.3389/fneur.2021.754518

**Published:** 2021-11-11

**Authors:** Marta Simone, Claudia Palazzo, Mariangela Mastrapasqua, Luca Bollo, Francesco Pompamea, Alessandra Gabellone, Lucia Marzulli, Paola Giordano, Andrea De Giacomo, Antonio Frigeri, Maddalena Ruggieri, Lucia Margari

**Affiliations:** ^1^Department of Biomedical Sciences and Human Oncology, School of Medicine, University of Bari Aldo Moro, Bari, Italy; ^2^Department of Basic Medical Sciences, Neurosciences and Sense Organs, University of Bari Aldo Moro, Bari, Italy

**Keywords:** serum neurofilament light chain, encephalopathy, myelin oligodendrocyte antibody, pediatric acute demyelinating disease, biomarkers

## Abstract

**Introduction:** The relationship between serum neurofilament light chain (sNfL) and myelin oligodendrocyte glycoprotein antibody (MOG-Ab) status has not been yet investigated in children with the acquired demyelinating syndrome (ADS).

**Objective and Methods:** The sNfL levels and MOG-Abs were measured by ultrasensitive single-molecule array and cell-based assay in a cohort of 37 children with ADS and negativity for serum anti-aquaporin 4 (AQP4) antibodies. The sNfL levels were compared in MOG-Ab+/MOG-Ab– and in two subgroups MOG-Ab+ with/without encephalopathy.

**Results:** About 40% ADS resulted in MOG-Ab+. MOG-Ab+ were younger at sampling (median = 9.8; range = 2.17–17.5 vs. 14.7/9–17; *p* = 0.002) with lower frequency of cerebrospinal fluid oligoclonal bands positivity (27% vs. 70%; *p* = 0.013) compared to MOG-Ab–. About 53% of MOG-Ab+ presented encephalopathy at onset, 1/22 of MOG-Ab– (*p* = 0.0006). Higher sNfL levels (*p* = 0.0001) were found in MOG-Ab+ (median/range = 11.11/6.8–1,129) and MOG-Ab– (median/range = 11.6/4.3–788) compared to age-matched controls (median/range = 2.98/1–4.53), without significant difference. MOG-Ab+ with encephalopathy resulted significantly younger at sampling (median/range: 4.5/2.17–11.17 vs. 14.16/9.8–17.5; *p* = 0.004), had higher sNfL levels (median/range:75.24/9.1–1,129 vs. 10.22/6.83–50.53; *p* = 0.04), and showed a trend for higher MOG-Ab titer (0.28/0.04–0.69 vs. 0.05/0.04–0.28; *p* = 0.1) in comparison to those without encephalopathy.

**Discussion:** We confirmed high sNfL levels in pediatric ADS independently from the MOG-Ab status. Encephalopathy at onset is associated more frequently with MOG Ab+ children with higher sNfL levels and MOG titer. These findings suggest a role of acute demyelination in association with axonal damage in the pathogenesis of encephalopathy in pediatric ADS.

## Introduction

The occurrence of the first event of acquired demyelinating syndromes (ADS) in children is estimated to be 0.87 (95% CI 0.35–1.40) per 100,000 children per year (1).

Anti-myelin oligodendrocyte glycoprotein antibodies (MOG-Abs) have emerged as potential biomarkers useful for the differential diagnosis and reflecting pathogenic mechanisms, disease course, prognosis, and therapeutic response in pediatric patients with ADS (2–6). Serum neurofilament light chain (sNfL) is a marker of neurodegeneration in several neurological diseases (7–10).

The NfL is a component of the neuron cytoskeleton and is released in the extracellular space after neuronal cell death (11, 12).

A significant correlation has been found between cerebrospinal (CSF) and sNfL levels in children with ADS (2) suggesting that sNfL could be a promising peripheral biomarker for axonal injury.

The MOG-Abs had been demonstrated to activate Ab-dependent cellular cytotoxicity against MOG, located on the myelin surface in the central nervous system, determining demyelination (13).

Recent data showed a correlation between clinical/radiological activity, cognitive impairment, and an increase of sNfL levels in pediatric patients with multiple sclerosis (MS) and clinically isolated syndrome (CIS) (2, 3). High sNfL levels were also found in patients with neuromyelitis optica spectrum disorders (NMOSDs) associated with aquaporin-4 (AQP-4) antibodies (3) and in patients with the anti-MOG-Ab-associated disorder (MOGAD) (14) indicating the presence of axonal damage in all these disorders. The overall incidence of MOGAD is 0.16 per 100,000 people, with higher seropositivity in children (0.31/100,000) than in adults (0.13/100,000), especially in patients who have experienced an ADS prior to the age of 10 years (15). The most common presenting phenotype of MOGAD is acute disseminated encephalomyelitis in children (40–56%) and optic neuritis (ON) in adults (44%) (16). The relationship between MOG-Ab titers and sNfL levels remains an area of active investigation. A recent paper (17) demonstrated in a series of 38 consecutive adult onset ADS MOG-Ab+ that sNfL levels correlated with attack severity and predicted long-term outcome. The same authors, (18) more recently, analyzed sNfL levels at onset and during follow-up in 18 adult ADS MOG-Ab+ and showed that sNfL levels predominantly increase at disease onset. To date, the relationship between sNfL levels and MOG-Abs has not yet been investigated in pediatric populations with ADS.

In this study, in a cohort of 37 children with the first event of ADS, we tested sNfL levels in MOG-Ab+ and MOG-Ab– patients, and in two subgroups of MOG-Ab+ subdivided into those with and without encephalopathy. Moreover, we compared demographic, clinical, neuroimaging, and laboratory features between these groups.

## Methods

### Study Population

Between February 2019 and December 2020, 37 patients (F/M 15/22) younger than 18 years with a first acute event of ADS and negativity for serum anti-AQP4 antibodies (AQP4 antibodies are routinely requested in all children with ADS in our center) were recruited at the Neuropsychiatric Unit and the Paediatric Neurology Unit of University/Hospital Policlinico of Bari. All patients underwent blood sampling at the time of the first presentation before starting any treatment.

All sera were tested for sNfL levels and for the presence and levels of MOG-Abs. An additional group of 20 serum samples from age-matched unaffected controls were also tested for sNfL. Baseline demographic (age and gender), clinical (symptoms at onset classified as ON, brainstem involvement, spinal cord involvement, encephalopathy; monofocal or multifocal onset), MRI (presence of brain and/or spinal cord abnormalities), and laboratory [CSF presence/absence of oligoclonal bands (OB)] features were recorded for all patients.

### Serum MOG IgG-Ab Assessment

The MOG-Abs were detected by a cell-based assay (CBA). Briefly, cultured human cells (HEK293 cells) were transfected with a vector coding for the antigen MOG conjugated to GFP and used as substrate in an indirect immunofluorescence assay (19). Immunofluorescence was performed on transfected cells cultured on glass coverslips. Cells were exposed to the sample sera for 1 h at room temperature, and serum MOG-IgG was detected on the surface of MOG expressing cells, using goat anti-human 568 Alexa-Fluor conjugated secondary antibodies (Invitrogen, Life Technologies, Carlsbad, CA, USA). In order to evaluate unspecific background staining, we routinely performed serum antibody staining using empty vector transfected cells for both immunoassays. A cover glass containing cells was observed using a DMRXA fluorescence microscope (Leica Microsystems, Wetzlar, Germany) provided with a DFC700T color camera. The antibody titer for MOG-Abs was performed by a ratiometric method (20) using the cells expressing the fluorescent protein (MOG-GFP). Briefly, for each cell line, the analysis was performed using a 20× objective, and two microscopy fields were randomly chosen. For each field, four different regions containing two to four cells were analyzed. Each region was analyzed, fluorescent intensity was measured with a grayscale, and background subtracted values were given as the ratio of red/green for the MOG antibody titer. Values range 0 for no staining and 1 for maximum antibody binding. Therefore, we defined these values as MOG quantitative ratio (MOGqr).

### Serum NfL Assessment

The sNfL levels were determined by an ultrasensitive single-molecule array (Simoa) method using the Quanterix-SRX Analyzer Platform (Quanterix, Lexington MA, USA). Blood samples were collected from each of the participants, centrifuged, and sera divided into aliquots were frozen at −80°C for subsequent use.

The sNfL concentrations were measured *via* the commercially available Simoa^TM^NF-light^®^ Advantage Kit (Quanterix, Lexington MA, USA), a Singleplex Assay Kit.

Samples (25 μl of serum was diluted 1:4 in the plate by adding 75 μl of sample diluent in each well) were tested blindly and in duplicate and two quality control (high concentration and low concentration quality control) samples were run, in duplicate as well, on each plate for each run necessary to complete the study. The sNfL concentrations (pg/ml) were calculated using a standard curve made from a sample of known NfL concentrations in triplicate according to the instructions of the manufacturer.

The intra-assay coefficient of variation (CV) values have been calculated by the SRX Analyzer Software (Quanterix Corp. Billerica Massachusetts, US) from technical replicate measures of specimens assayed within a single run.

### Statistical Analysis

Statistical analysis was performed by using IBM SPSS, release v.20.0 (IBM Corporation, Armonk, NY, USA). We compared demographic, clinical, neuroimaging, and serological data by the Kruskal–Wallis test, Mann–Whitney test, Wilcoxon rank test, Fisher's exact test, and χ2 test. Correlation between age at sampling and sNfL levels were evaluated by the Spearman test. Statistical significance was defined as a two-sided *p* < 0.05.

## Results

About 15 ADS (40%) resulted MOG-Ab+ and 22 (60%) MOG-Ab–. The median value and range of MOG-Ab was 0.12/0.04–0.69 MOGqr in MOG-Ab+ patients. Demographic, clinical, laboratory, and radiological data of the two cohorts are reported in Table 1. MOG-Ab+ ADS had a significantly lower median age at sampling (median = 9.8; range = 2.17–17.5 vs. 14.7/ 9–17; *p* = 0.002) and showed a lower frequency of CSF OB positivity (27% vs. 70%; *p* = 0.013) in comparison to MOG-Ab– ADS. About 53% of MOG-Ab+ patients presented encephalopathy at onset, but only in 1/22 of MOG-Ab– patients (*p* = 0.0006). A multifocal onset was associated more frequently (53%) in MOG-Ab+ than in MOG-Ab– (27%; *p* = 0.09) ADS.

**Table 1:** Comparison of baseline demographic, clinical, laboratory and radiological features between pediatric ADS MOG-Ab positive (+) and MOG-Ab negative (–).

	**ADS** **MOG-Ab+**	**ADS** **MOG-Ab-**	***p*** **-value**
Number	15	22	
Female, *n* (%)	5 (33%)	9 (40%)	ns
Age at sampling, median (range), years	9.8/2.17–17.5	14.7/9–17	0.002
MOG-Ab titre, median (range)	0.12/0.04–0.69	/	
CSF IgG oligoclonal bands (%)	4/15 (27%)	15/22 (70%)	0.013
Abnormal brain MRI (%)	11/15 (75%)	19/22 (87%)	ns
Abnormal spinal cord MRI (%)	9/22 (62%)	13/22 (59%)	ns
Encephalopathy at onset	8/15 (53%)	1/22 (4.5%)	0.006
Optic Neuritis at onset (%)	7/15 (47%)	11/22 (50%)	ns
Myelitis at onset (%)	8/15 (53%)	14/22 (64%)	ns
Brainstem at onset (%)	7/15 (47%)	7/22 (32%)	ns
Multifocal onset	8/15 (53%)	6/22 (27%)	0.09

Among MOG-Ab– patients who had CSF OB positivity (15/22), nine of them had MRI features typical or suggestive of MS, and these patients fulfilled criteria for a CIS diagnosis (21).

**Figure 1:**
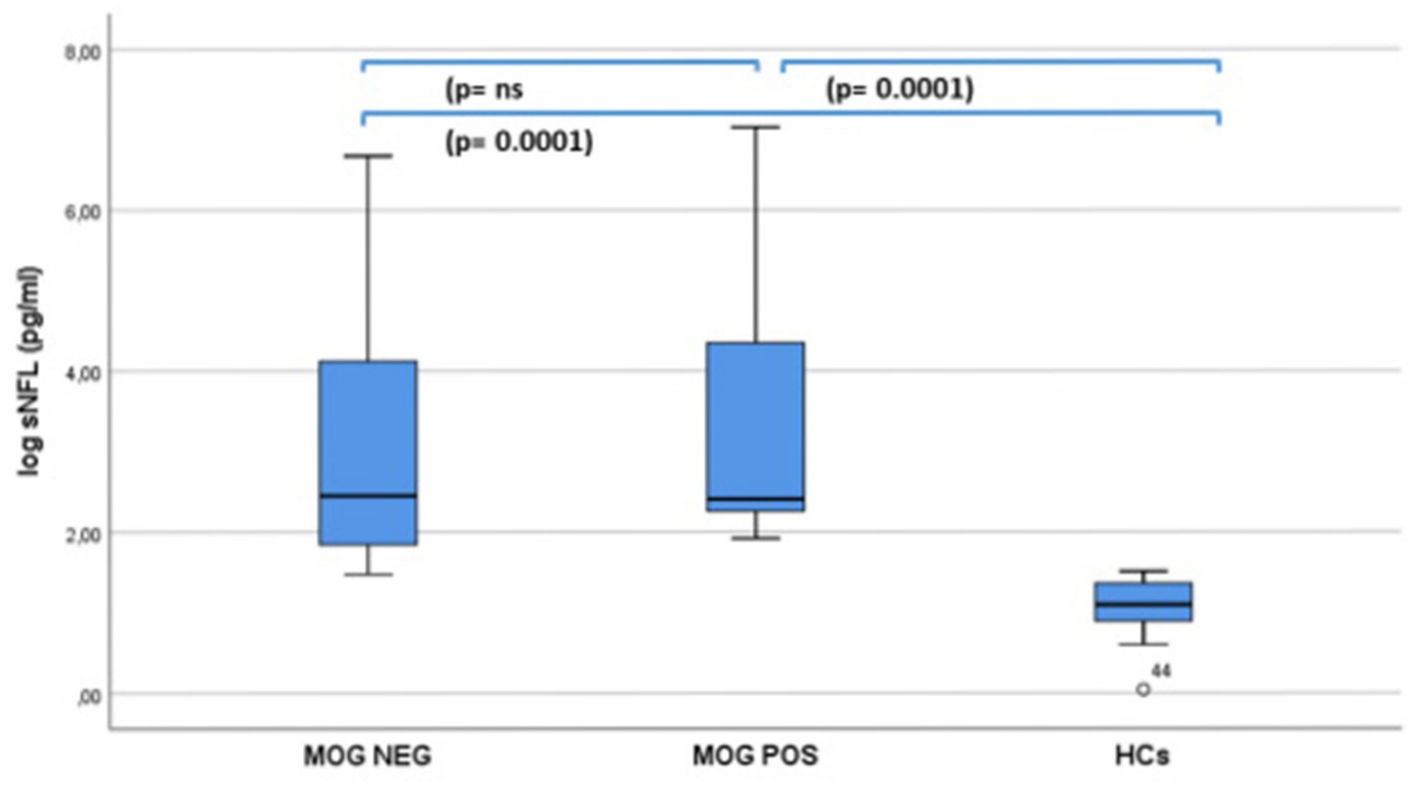
sNF-L levels in 15 MOG-Ab positive and 22 MOG-Ab negative children with a first acute event of acquired demyelinating syndrome and 20 age-matched health controls (I-ICs).

**Figure 2:**
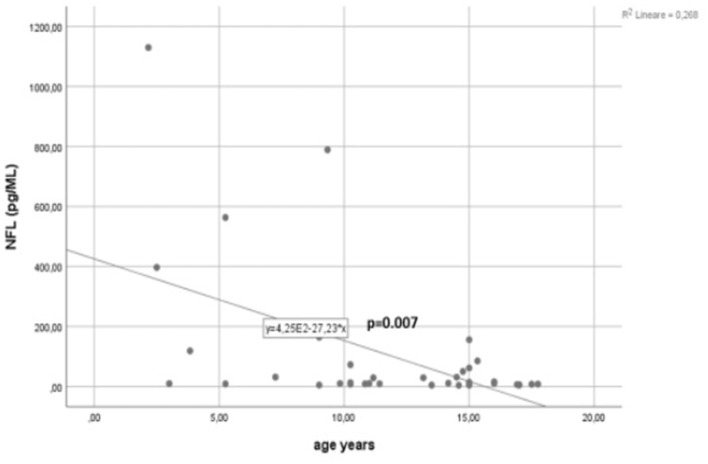
Correlation between age at sampling and sNFL levels in 37 children with a first acute event of acquired demyelinating syndrome (ADS).

Significant (*p* = 0.0001) higher sNfL levels were found in both MOG-Ab+ (median/range = 11.11/6.8–1,129) and MOG-Ab– (median/range = 11.6/4.3–788) patients compared to age-matched controls (median/range = 2.98 (1–4.53), but no significant difference was found between the two groups (Figure 1). A significant negative correlation was found between age at sampling and sNfL levels in this cohort of pediatric ADS (*R* = −0.434, *p* = 0.007) (Figure 2).

When compared MOG-Ab+ patients with (N. 8) and without (N.7) encephalopathy (Table 2), children belonging to the first group resulted significantly younger at sampling (median and range: 4.5/2.17–11.17 vs. 14.16/9.8–17.5; *p* = 0.004), had higher sNfL levels (median/range: 75.24/9.1–1,129 vs. 10.22/6./6.83–50.53; *p* = 0.04), and showed a trend for higher MOG-Ab titers (0.28/0.04–0.69 vs. 0.05/0.04–0.28; *p* = 0.1) in comparison to those without encephalopathy.

**Table 2:** Comparison of baseline demographic and laboratory features between MOG-Ab positive (+) ADS with encephalopathy and MOG-Ab positive (+) ADS without encephalopathy.

	**ADS** **MOG+ without Encephalopathy**	**ADS** **MOG+ with Encephalopathy**	***p*** **-value**
Number	7	8	
Gender (F/M)	2/7	3/8	ns
Age at sampling, median (range), years	14.16 (9.8–17.5)	4.5 (2.17–11.17)	0.004
MOG-Ab titre, median (range)	0.05 (0.04–0.28)	0.28 (0.04–0.69)	0.1
sNfL median (range) pg/ml	10.22 (6.83–50.53)	75.24 (9.1–1.129)	0.043

**Figure 3:**
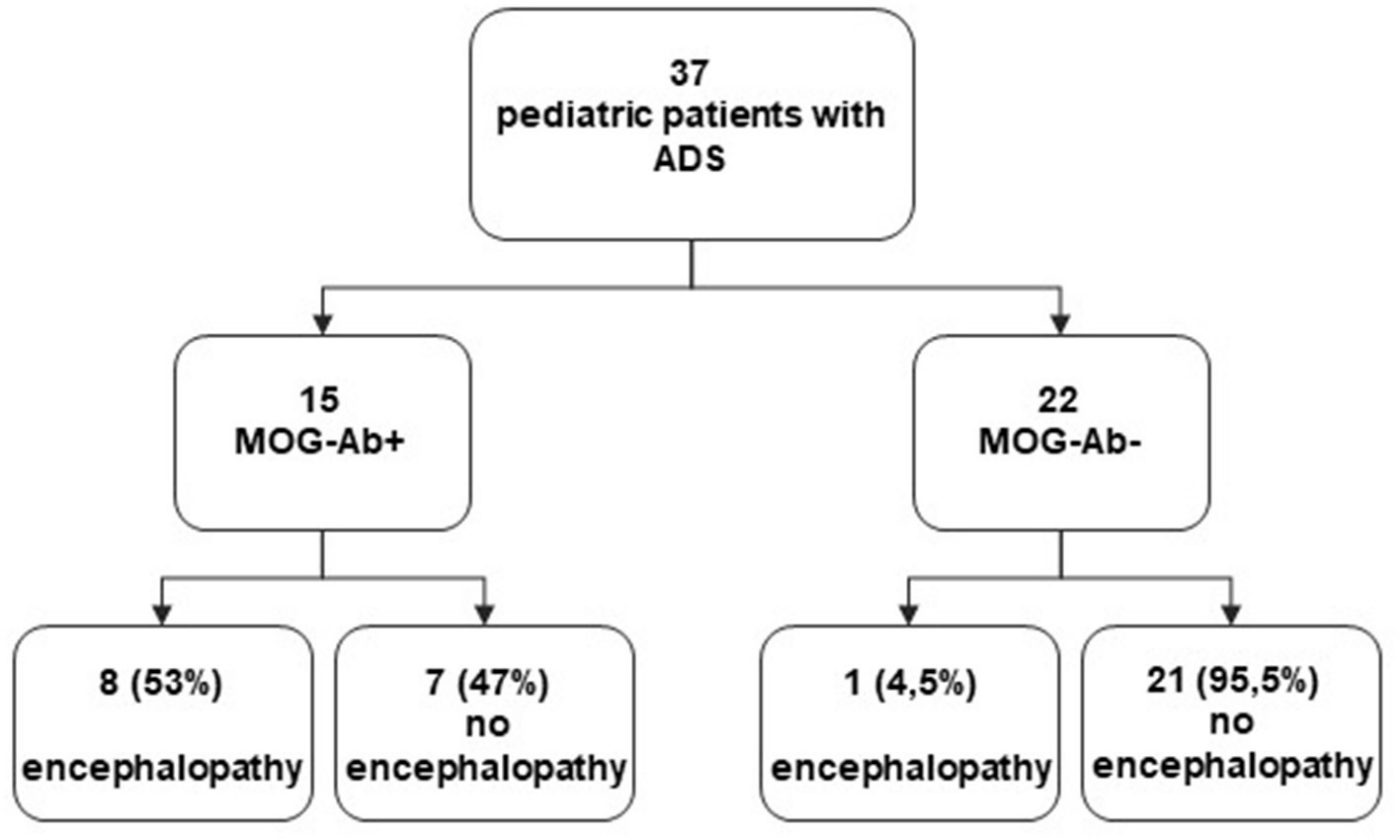
Flowchart of the study population.

Figure 3 represents a flowchart for the whole study population.

## Discussion

In this study, we assessed for the first time in a pediatric population with a first acute event of ADS, sNfL levels according to MOG-Ab status. By using a sensitive MOG-Ab assay, we identified 40% of MOG-Ab+ patients in a selected cohort of pediatric ADS confirming the results observed in previous similar cohorts (22), showing that 30–40% of pediatric ADS were associated with MOG-Ab positivity. MOG-Ab+ children were younger at onset in comparison to the MOG-Ab– patients as reported in a previous study in which about 50% of MOG-Ab+ had <11 years at presentation (15). We found a lower frequency of CSF OB positivity (27%) in MOG-Ab+ ADS patients in comparison to MOG-Ab– patients (70%) as found in a previous larger population (23) showing a frequency of 9.1% of CSF OB positivity in MOG-Ab+ and of 94.6% in MOG-Ab– pediatric MS patients. The frequency of the abnormal brain and spinal cord findings did not differ between the two groups. According to previous studies showing a majority of cases (50%) of monophasic forms of MOGAD manifest as acute disseminated encephalomyelitis, especially in patients under the age of 5–10 years (22, 24, 25), in our study, we found encephalopathy was present at onset in 53% of MOG-Ab+ patients and only 1/22 MOG-Ab– (<1%), and MOG-Ab+ patients with encephalopathy resulted significantly younger at sampling than MOG-Ab+ patients without encephalopathy. sNfL levels did not differ between pediatric MOG-Ab+ and MOG-Ab– children, but they resulted significantly higher in both groups compared to age-matched controls, confirming that neuroaxonal damage is a prominent feature in ADS. A significant negative correlation was found, in our cohort of pediatric ADS, between age at sampling and sNfL levels. Earlier studies observed an association between sNfL and age, with higher sNfL levels in younger pediatric MS, and a correlation between age and CSF NfL with the highest levels in younger children with neurologic diseases (26). This finding is consistent with histologic observations for pediatric MS lesions, where the amount of acutely damaged axons inversely correlated with the age of patients (27). A recent paper (2) demonstrated in a cohort of 102 pediatric patients with a first acute event of ADS that sNfL levels were higher in children presenting with encephalopathy than in those without encephalopathy symptoms. In the current study, we further demonstrated that in ADS MOG-Ab+ children, encephalopathy is associated with higher sNfL levels and a trend for higher MOG titer. These findings not only confirm the prominent role of axonal damage in the pathogenesis of encephalopathy in pediatric MOGAD (28) but also suggest a possible associated role of acute demyelination.

The role of local anti-MOG IgG deposits and complement activation in the pathogenesis of EAE and ADS MOG+ is still a matter of debate. Serguera et al. (29) reported that IgG and C1q were associated with myelin and phagocytic cells in brains with EAE and in biopsies of children with ADS MOG+, suggesting that local anti-MOG IgG deposits and complement activation in the perivenular white matter may play a central role in the pathogenesis of EAE and ADS MOG+, initiating and amplifying demyelination. Another paper (30) demonstrated that MOG-Abs from most patients require bivalent binding that poorly binds to C1q, questioning the pathogenicity of MOG-Abs is mediated by complement activation. In a more recent study (28) conducted on immunohistochemically analyzed brain tissue biopsies from 11 patients with MOG-Ab-associated disease and other inflammatory demyelinating diseases, the occurrence of perivascular deposits of activated complements and immunoglobulins was occasionally observed in some MOG-Ab-associated demyelinating lesions even if the frequency was much lower than that in AQP4 antibody-positive NMOSD.

These discordant results indicate the need for further studies to ascertain the role of MOG-Abs in the pathogenesis of MOGAD.

The main limitation of this study concerns the small size of each group, especially the subgroup of ADS MOG+; nonetheless, we provide convergent observations with other larger studies. Clearly, larger cohorts and longitudinal studies in pediatric ADS are needed to better investigate the potential interactions between MOG-Ab titers and sNfL levels.

## Data Availability Statement

The raw data supporting the conclusions of this article will be made available by the authors, without undue reservation.

## Ethics Statement

The studies involving human participants were reviewed and approved by Policlinico of Bari Ethical Committee. Written informed consent to participate in this study was provided by the participants' legal guardian/next of kin.

## Author Contributions

MS and LM had full access to all of the data in the study and take responsibility for the integrity of the data and the accuracy of the data analysis. MS, LM, AF, and MR study concept and design. MS, FP, LM, and AD drafting of the manuscript. MS, LM, LB, AF, and MR critical revision of the manuscript for important intellectual content. LB statistical analysis. MS, FP, AG, and LM administrative, technical, or material support. All authors acquisition, analysis, or interpretation of data.

## Conflict of Interest

The authors declare that the research was conducted in the absence of any commercial or financial relationships that could be construed as a potential conflict of interest.

## Publisher's Note

All claims expressed in this article are solely those of the authors and do not necessarily represent those of their affiliated organizations, or those of the publisher, the editors and the reviewers. Any product that may be evaluated in this article, or claim that may be made by its manufacturer, is not guaranteed or endorsed by the publisher.
